# MassARRAY-based single nucleotide polymorphism analysis in breast cancer of north Indian population

**DOI:** 10.1186/s12885-020-07361-8

**Published:** 2020-09-07

**Authors:** Divya Bakshi, Ashna Nagpal, Varun Sharma, Indu Sharma, Ruchi Shah, Bhanu Sharma, Amrita Bhat, Sonali Verma, Gh. Rasool Bhat, Deepak Abrol, Rahul Sharma, Samantha Vaishnavi, Rakesh Kumar

**Affiliations:** 1 School of Biotechnology, Shri Mata Vaishno Devi University, Katra, Jammu and Kashmir India; 2 Ancient DNA Laboratory, Birbal Sahni Institute of Palaeosciences, Lucknow, Uttar Pradesh India; 3 Department of Radiotherapy, GMC, Kathua, J&K India; 4 Department of Radiotherapy, GMC, Jammu, J&K India; 5 Department of Plant Sciences, Central University of Jammu, Jammu, J&K India

**Keywords:** Breast cancer, Single nucleotide polymorphism, Jammu and Kashmir

## Abstract

**Background:**

Breast Cancer (BC) is associated with inherited gene mutations. High throughput genotyping of BC samples has led to the identification and characterization of biomarkers for the diagnosis of BC. The most common genetic variants studied are SNPs (Single Nucleotide Polymorphisms) that determine susceptibility to an array of diseases thus serving as a potential tool for identifying the underlying causes of breast carcinogenesis.

**Methods:**

SNP genotyping employing the Agena MassARRAY offers a robust, sensitive, cost-effective method to assess multiple SNPs and samples simultaneously. In this present study, we analyzed 15 SNPs of 14 genes in 550 samples (150 cases and 400 controls). We identified four SNPs of genes *TCF2*1, *SLC19A1, DCC,* and *ERCC1* showing significant association with BC in the population under study.

**Results:**

The SNPs were rs12190287 (*TCF2*1) having OR 1.713 (1.08–2.716 at 95% CI) *p*-value 0.022 (dominant), rs1051266 (*SLC19A1*) having OR 3.461 (2.136–5.609 at 95% CI) *p*-value 0.000000466 (dominant), rs2229080 (*DCC*) having OR 0.6867 (0.5123–0.9205 at 95% CI) p-value 0.0116 (allelic) and rs2298881 (*ERCC1*) having OR 0.669 (0.46–0.973 at 95% CI), p-value 0.035 (additive) respectively. The *in-silico* analysis was further used to fortify the above findings.

**Conclusion:**

It is further anticipated that the variants should be evaluated in other population groups that may aid in understanding the genetic complexity and bridge the missing heritability.

## Background

Cancer is a neoplastic disease consisting of cancer cells that harbor numerous biological capabilities that occur due to the accumulation of various genetic aberrations and genomic alterations [1]. In 2018, about 18 million new cases of cancer were recorded and about 9.5 million deaths occurred with lung and BC; being the leading cause of mortality in men and women, respectively [2]. BC is the leading cancer of women in India, with 27.7% of all the cancers in women [3]. A study conducted in the Kashmir province of Jammu and Kashmir highlighted BC to be the second most common cancer in women with 16.1% of all the cancers, closely following colorectal cancer (16.8%) [4]. Despite the exacerbating rate of BC in J&K, there is a high susceptibility of post-menopausal women to develop breast cancer, especially with numerous factors underplay [5, 6]. Early menarche and late menopause ensure a longer exposure to the hormone estrogen thus increasing the risk of BC [7]. Various studies have identified the variations in high penetrant genes like *BRCA1, BRCA2*, *PTEN*, *TP53*, *CDH1,* and *STK11* along with moderate penetrant genes such as *CHEK2*, *BRIP1*, *ATM* and *PALB2* [8, 9] and their association with BC. There are about 182 loci that have been identified and are susceptible to BC [10] which accounts for 30% of the genetic heritability of BC [11]. Keeping in view the missing heritability and scanty literature of BC in studied population group, we investigated cancer-associated variants [12, 13] to get an insight into their role in BC development among the population of Jammu and Kashmir. With an effort to bridge this gap, we conducted a case-control association study in population of post-menopausal women from North India.

## Methods

### Sample collection and DNA isolation

Sampling was carried out from January 2015 to December 2018. A total of 550 age-matched samples (150 cases and 400 controls) were recruited for the study. The cases were histo-pathologically confirmed with no evident history of any other cancer. The Institutional Ethical Review Board (IERB) (SMVDU/IERB/18/70) of Shri Mata Vaishno Devi University approved this study. After written informed consent, blood samples, about 2 ml of venous blood was collected into EDTA vacutainers from patients and healthy controls (age and ethnicity matched). Qiagen DNA isolation kit (Catalogue number 51206) was employed for the genomic DNA isolation from a whole blood sample, using the manufacturer’s protocol. Quality check was performed using 0.8% gel electrophoresis and was quantified using the Eppendorf India Pvt. Ltd. Bio-spectrophotometer™.

### SNP genotyping

Agena Bioscience MassARRAY iPLEX GOLD chemistry technology was used to perform genotyping. Primers flanking the gene region of the SNPs were designed in the AgenaCx platform (GRCh38/hg38) (https://agenacx.com/). DNA sample with a concentration of 10 ng/μl was used with volume 1 μl for the PCR reaction. The first PCR was performed using forward and reverse primer pool and shrimp alkaline phosphatase was then used to neutralize unincorporated dNTPs from the first PCR reaction. iPLEX extension reaction was performed using pool single base extension primers. Desalting of the amplified products was performed using SpectroCLEAN resin following the protocol [14, 15]. RS100 nanodispenser was used to transfer the cleaned extension products from 384 muti-titration PCR plate to 384 SpectroCHIP and the chip was placed in Agena MassARRAY compact mass spectrometer. A spectrum of the different products was acquired by SpectroAcquire software. Data in the form of genotypes and call clusters were obtained using Agena MassARRAY Typer V4.0.5. The representative workflow of genotyping has been summarized in Fig. S1 (Supplementary Figure).

### Candidate gene and SNP selection

The genes selected for the present study are involved in following cancer-related pathways: Tumor suppressor, DNA damage and repair, regulation of cell proliferation, telomere maintenance, cell cycle regulation, and apoptosis. Variations with a potential functional effect like causing amino acid alteration, present in the promoter and UTR region, having an effect on splicing site and transcription binding sites were also selected for the study. The potential genetic variants were retrieved from the database. Variations with an allelic frequency greater than 5% in Gujarati Indians from Houston (GIH) and Western European Ancestry (CEU) as it has been observed that genetic makeup of the studied population is similar to Europeans [16]. The studied genes and variants with their putative role and annotations are summarized in the Table S3.

### Statistical analysis

Chi-square analysis was performed by using the Plink V.1.09 to observe the allelic association between cases and controls [17]. Further, the association analysis was performed with different genetic models, and the association was calculated in terms of Odds Ratio (OR) at 95% confidence interval (CI) using Statistical Package for the Social Sciences (SPSS) version 20. The corrected OR was obtained using unconditional logistic regression analysis with age and, BMI as confounding factors. Further, the prediction of mRNA secondary structure of the variants have been illustrated by RNA fold [18] and minimum free energy (MFE) calculated from RNA fold was used to measure the stability of the secondary structures of mRNAs. The gene-gene interaction was performed using Cytoscape version 3.8.0 [19] by installing the GeneMANIA plugin [20]. The allele frequencies were compared with the global database 1000 genome [21] using a web-based application LD link [22] to observe the genetic heterogeneity in allele frequencies among different population groups.

## Results

### Genetic variants associated with the increased risk of BC in the population under study

Out of the fifteen variants shortlisted four variants namely rs1051266, rs12190287, rs2229080, and rs2298881 in *SLC19A1*, *TCF21*, *DCC*, and *ERCC1* genes respectively, were found to be significantly associated with BC in the studied cohort. The variant rs1051266 is located in the second exon of the gene *SLC19A1* and is a missense variant. The variant shows a significant association with BC with OR 1.745 (1.321–2.304 at 95% CI) and has a *p* value = 7.98E-05 (Allelic). A significant association of the variant was also observed in the dominant model with OR 3.461 (2.136–5.609 at 95% CI) and p value = 0.000000466 (Table 1). The variant was providing risk for BC in the studied cohort. The variant rs12190287 is a 3’UTR variant located in the third exon of the gene *TCF21*. The allelic association of the variant showed a weak association with BC and OR observed was 1.306 (0.995–1.713 at 95% CI) having *p*-value = 0.0491 (Allelic) to observe the maximum effect of allele C, the dominant model was evaluated. Interestingly, the OR observed was 1.713 (1.08–2.716 at 95% CI) and *p* value = 0.022 (Table S2). The variant was providing risk for BC in the dominant model in the studied cohort. The variant rs2298881 is located at the intron of the *ERCC1* gene. Variant rs2298881 was found significantly associated with BC and the OR observed was 0.6981 (0.36–0.71 at 95% CI) and p value = 0.01169 (Allelic). A significant association was observed with the additive model with OR 0.669 (0.46–0.973 at 95% CI), *p*-value = 0.035 (Table S2). The variant rs2229080 is a missense variant, located on the third exon of the *DCC* gene. The variant rs2229080 showed protection against BC having OR 0.6867 (0.5123–0.9205 at 95% CI) and *p* value = 0.011 (Allelic). However non-significant association was observed for rs2229080 using the dominant model: OR 0.797 (0.517–1.229 at 95% CI), *p*-value = 0.305, recessive model: OR 0.505 (0.252–1.014 at 95% CI), p value = 0.05 and Additive model: OR 0.758 (0.551–1.042 at 95% p-value = 0.088 (Table S2).

**Table 1:** Logistic regression analysis of the variants in the studied population group

S.No.	GENE	SNP	CASES	CONTROL	H.W.E.	OR AT 95% CI	p-VALUE
1	*TCF21*	rs12190287	C = 0.45	C = 0.39	0.456	1.306 (0.995–1.713)	0.0491
			G = 0.55	G = 0.61			
2	*DCC*	rs2229080	C = 0.38	C = 0.47	0.111	0.6867 (0.5123–0.9205)	0.01169
			G = 0.62	G = 0.53			
3	*SLC19A1*	rs1051266	T = 0.46	T = 0.33	0.053	1.745 (1.321–2.304)	7.98E-05
			C = 0.54	C = 0.66			
4	*ERCC1*	rs2298881	A = 0.21	A = 0.27	0.124	0.6981 (0.36–0.71)	0.03043
			C = 0.79	C = 0.73			

### Genetic variants not associated with BC in the population under study

The intronic variant rs249954 on the *PALB2* gene has been found associated with the breast cancer risk in Chinese population, however has no association with breast cancer in the population under study [23]. The variant rs664677 in the DNA damage response gene *ATM* though not associated in our population, previously has been reported to be associated with breast cancer and lung cancer risk in Asian people [24]. Another variant rs2981582, in the *FGFR2* gene, which was not associated in the population under study, was found associated with breast cancer in Asian and Caucasian population groups [25]. The gene *SLC4A7* having the rs4973768 although not significantly associated with breast cancer in our population previously has been found associated with increased risk of breast cancer in Chinese population [26]. The variant rs2363956 of the *ANKLE-1* gene has been previously reported to be associated with breast cancer risk in Chinese population [27], however its role in the population under study remains ambiguous since only variants with call rate over 90% were acknowledged in this study. The gene *CYP19A1* with the variant rs10046 has previously been reported to be associated with estradiol levels and postmenopausal breast cancer in European population [28]. The 2 variants in *TERT* gene, rs2736100 and rs2735940, have been reported to be associated with multiple cancers (including breast etc.) in Asian and Caucasian population [29] and lung cancer risk [30] especially in Caucasian population, respectively. The variant rs2975843 in *TERF1* gene was not found associated with breast cancer in the population under study however has been reported to be associated with colorectal cancer in European descent population [31]. Studies have shown the *BRIP1* gene variant rs4986764 associated with breast cancer in Chinese population [32], however no significant association was found in the studied population. The variant rs3792152 in *REV1* gene, not associated with population under study, has been previously reported to be associated with the development of epithelial ovarian cancer in European population [33].

### Prediction of mRNA secondary structure

**Fig. 1:**
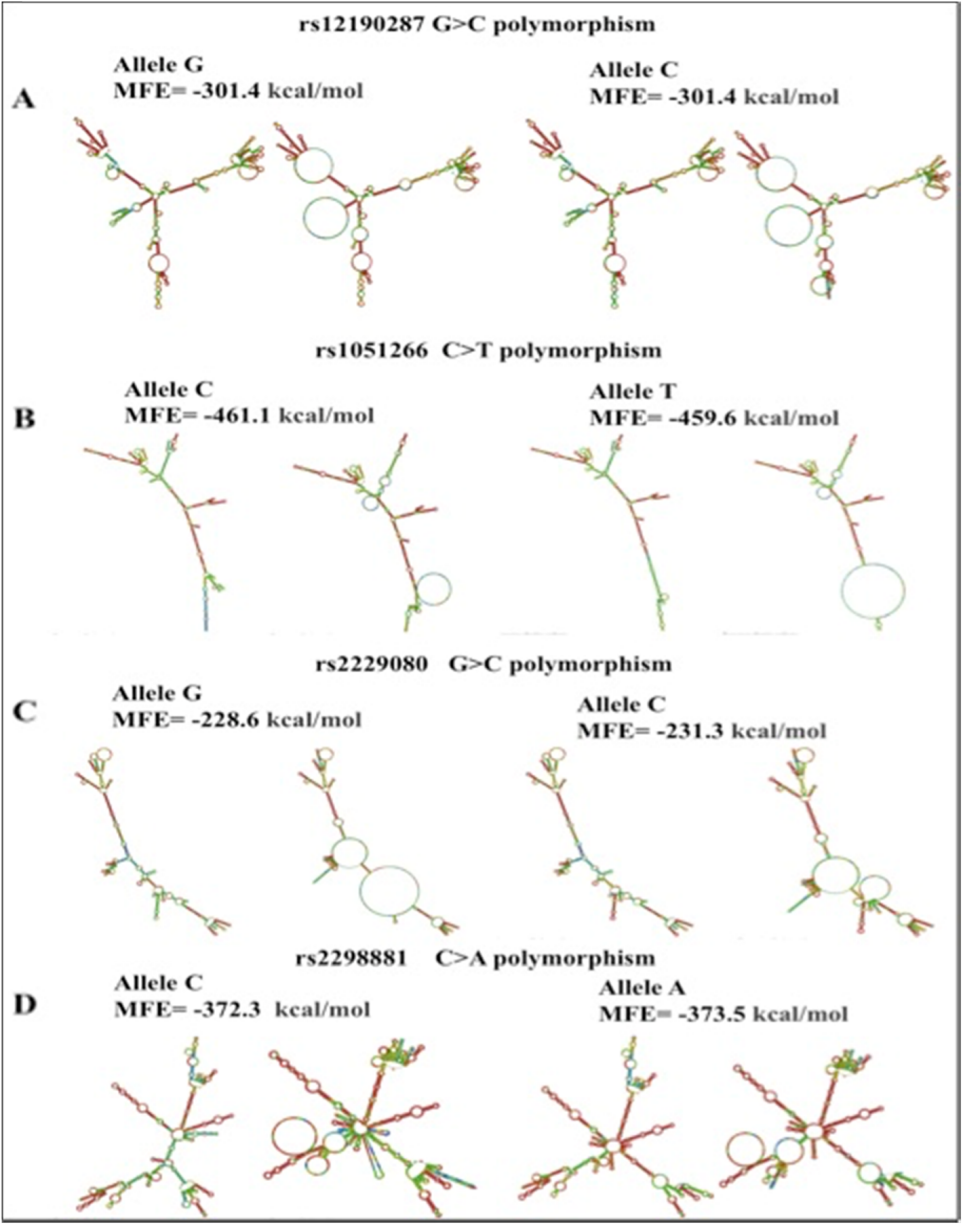
The predicted MFE and MFE centroid secondary structures of RNA and energies that were calculated by using RNA fold

MFE (Minimum free energy) secondary structure and the centroid secondary structure of the variants were studied (Fig. 1). The secondary RNA secondary structures of rs2229080, rs10,51,266, and rs2298881 polymorphisms revealed a slight variation in the energy of the wild type allele in comparison to the variant type. The variant rs1051266 had MFE of − 461.1 (Kcal/Mol) for the ancestral allele C, however, we observed an elevation in the MFE for the altered allele T being at − 459.6 (Kcal/Mol). Also, there was an increase in the MFE of centroid structure for this variant from − 372.57(Kcal/Mol) for allele C to – 372.57 (Kcal/Mol). For the variant rs12190287, no change was observed in the MFE value of the ancestral (G) and altered allele (C) structures, although there was an increase in the MFE of the centroid structure from − 234.28 (G) to − 222.46 (C) (Kcal/Mol). The variant rs2298881 was showing a decrease in the MFE from − 372.3(Kcal/Mol) for ancestral allele C to − 373.5(Kcal/Mol) for altered allele A. The MFE of the centroid structure was also found to be less for the wild allele C at − 277.15(Kcal/Mol) than the altered allele A at − 308.95 (Kcal/Mol). A significant change in the MFE value was observed for the variant rs2229080, the wild allele G being at − 228.6 (Kcal/Mol), and 231.1(Kcal/Mol) for the altered allele C. Their centroid structure MFE values also followed the same pattern and varied from being − 141.7 (Kcal/Mol) to − 164.3 (Kcal/Mol) for the wild and altered allele respectively. The decreased free energy of the wild allele correlates with increased stability in the structure. The observed decrease in MFE of the wild type allele for the variants rs1051266 and the MFE of the centroid structure for the variant rs12190287 corresponds with the increased stability of the wild type allele in both the cases. The altered allele of both the variants has been observed to risk causing. The decrease in the stability of the altered structure might be a potent factor posing the risk threat. The altered alleles of the variants rs2298881 and rs2229080 have a low MFE than the wild allele and have been shown for protecting the BC. The low MFE of the altered alleles confers greater stability to these structures than the wild alleles with a higher MFE value. The free energy values have been summarized in the Table S4.

### Network analysis

**Fig. 2:**
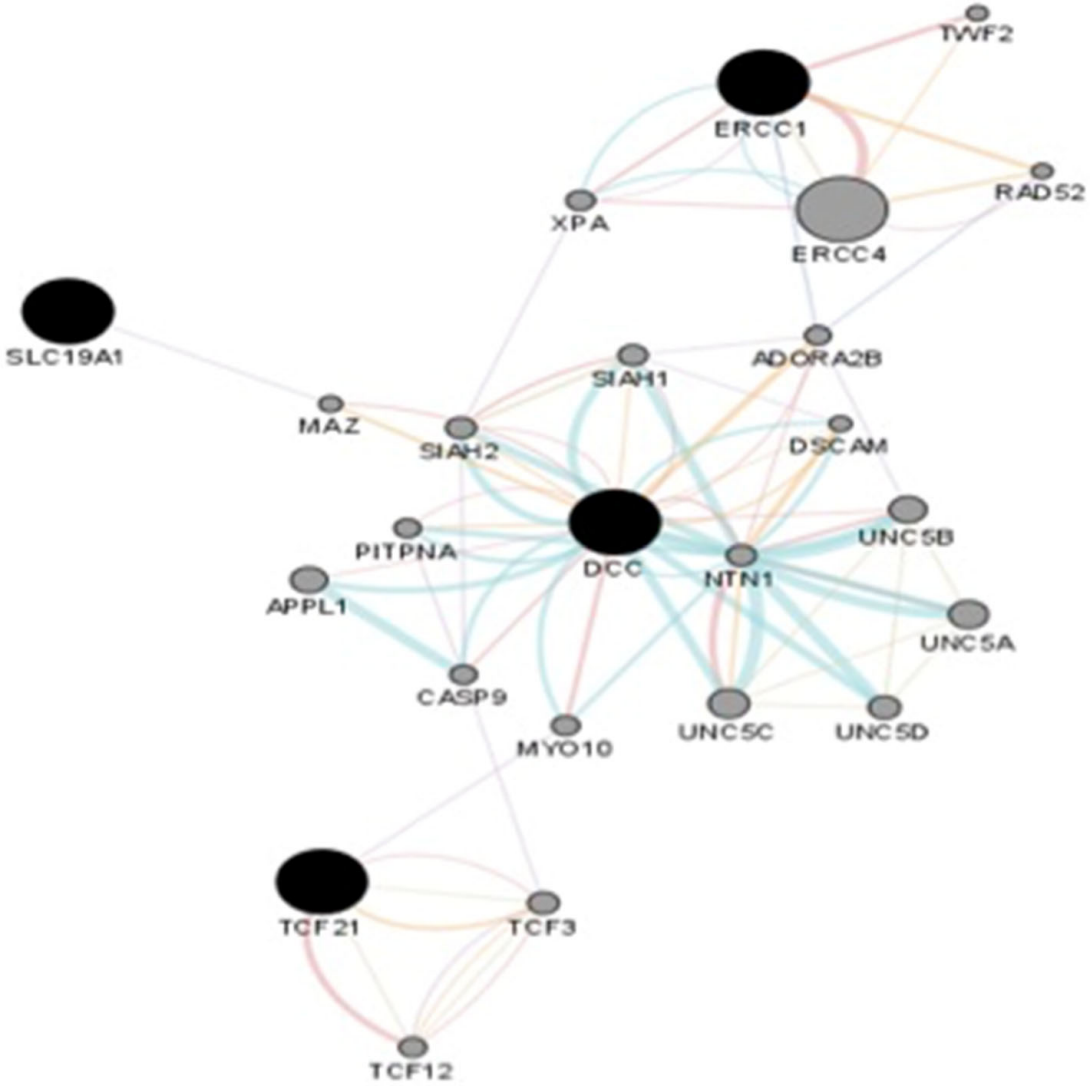
A network analysis of the associated genes using GeneMANIA database

The potential involvement of *DCC1*, *ERCC1*, *SLC19A1,* and *TCF21* relevant genes by querying the genes in the GeneMANIA [20] (Fig. 2). This showed that the expression of the genes is correlated with that of *DCC*. Further network analysis revealed that the *DCC* gene displays protein-protein interaction with *CASP9* that is associated with multiple cancer risks [34]. An interaction, however meek, is seen among the *DCC* interacting proteins and *TCF21*. No associated network of interacting proteins was found interacting with *SLC19A1*.

## Discussion

A replicative case-control study was done in about 550 samples to analyze the variants using Agena MassARRAY genotyping for the population of Jammu & Kashmir. Here, we investigate various BC loci in cases and controls. We investigated using the Agena massARRAY platform and identified numerous SNPs that were found significantly associated with BC genome-wide and independent of each other. Our study demonstrated 4 genome-wide loci which have been associated with BC development in the population under study. The rsIDs rs12190287 and rs1051266 associated with the genes *TCF21* and *SCL19A1* are causing risk in our population group. We also found six variants following HWE however not showing significance with BC development. The allele frequency of all the variants is shown in Figs. S2-S11.

The variant rs1051266 is located on the *SLC19A1* gene. *SLC19A1* or Solute Carrier family member protein is a gene implicated in placental carcinomas and pediatrics osteosarcomas. Studies have shown the *SLC19A1* gene variants to be associate with BC risk in worldwide populations [35] including African American women [36]. Our data revealed the variant rs1051266 to be significantly associated with BC risk in the population under study. Further, the bioinformatic analysis revealed that the associated variants are conserved in primates including humans and have been located in the conserved domain region (Fig. S13). We also studied the genotype tissue expression of the variants (GTEx) with their NES (Normalized Effect Size) values, which have been shown in Table S3. The GTEx with NES (Normalized Effect Size) was used to study the correlation between the genetic variation and gene expression in the human tissues. The variant 1,051,266 (*SLC19A1*) was significantly showing expression in breast tissue with an NES value of − 0.4333 and a *p*-value of 2.4e-6 (< 0.05).

*TCF21* or Transcription factor gene is a tumor suppressor gene and is associated with Uterine Corpus carcinoma and Pericoronitis. *TCF21* is found mutated in several types of cancers [37] Studies have shown a lower expression of *TCF21* in breast tumor tissues corresponding to enhanced tumor size and increased lymph node metastasis [38]. We analyzed the variant rs12190287 G > C of the *TCF21* gene and found it to be significantly associated with BC in the studied population group. The variant was found causing risk for BC in the population. The variant rs12190287 (*TCF21*) showed significant expression in breast tissue, with an NES value of 0.210 and a *p*-value of 3.3e-5. The positive NES value indicated the up-regulated expression in the breast tissue.

*ERCC1* gene or Excision Repair Cross-Complementing Rodent Repair gene which harbored the rs2298881 variant, functions in a nucleotide excision repair pathway [39]. *ERCC1* is found to be associated with multiple cancers. *ERCC1* variants have also been linked to an increased risk of BC [40] in women. The variant rs2298881 C > A was found significantly associated with breast cancer. The variant was found to be conferring protection for our BC in the studied population group. The variant rs2298881 (*ERCC1*) showed significant expression in the breast with an NES value of − 0.260 and a *p*-value of 3.8e-9.

*DCC* or Deleted in Colorectal Cancer is a gene encoding the netrin1 receptor. Netrin1 receptor is a transmembrane receptor belonging to the immunoglobulin superfamily. *DCC* gene is a tumor suppressor gene and is frequently mutated in colorectal carcinomas. *DCC* is abundantly expressed by neurons and stimulates cell survival and axon regeneration. Apart from mutations in colorectal cancers, studies have highlighted the role of *DCC* in BC. A variant of the *DCC* gene, rs2229080, has been found associated with increased BC risk [13]. Our study revealed that rs2229080 G > C was significantly associated with breast cancer and the altered allele C was causing protection in the studied population group. Though for the variant rs2229080 (*DCC*) the expression in breast tissue was found non-significant with the NES value of 0.054 with a *p*-value of 0.3. The positive NES value in rs12190287 (*TCF21*) is indicative of the up-regulation of the expression in the breast tissue and the variants 1,051,266 (*SLC19A1*) and rs2298881 (*ERCC1*) with negative NES points towards down-regulated expression in the breast tissue.

The RNA fold analysis revealed the MFE and structural differences in the wild and the altered allele. We also studied the difference in the secondary structures and the MFE values of the wild type allele and the variant allele. There was a decreased MFE in the case of the wild type allele of the variants rs12190287 and rs1051266 providing them an enhanced stable structure than the altered allele. Whereas, the rsIDs rs2229080 and rs2298881 associated with the genes *DCC* and *ERCC1* were found to be causing protection to BC. The MFE values of these variants were lower for the altered allele thus suggesting a more stable structure of these allele variants. The decrease in the MFE of the altered allele points towards an increase in the stability of the secondary structure. These variants have been found to be conferring protection for breast cancer in the studied population. Previously studies have elaborated on the codon selection biasness for a higher negative free energy and folding stability of the RNA secondary structure [10]. Owing to the myriad role of RNA structure in cancer development [11], it might be a potential cancer development risk in the population. Further analysis of the second structure of the genes with the variants highlighted a substantial difference in the MFE and MFE of centroid secondary structure. The differences in the MFE values of the variants have been summarized in the Table S4. The differences in the secondary structures of the alleles have been shown in Fig. 1. On comparing the allele frequencies of the associated allele with 1000genome data, we found substantial differences in the allele frequencies. The differences in the allele frequency of the associated alleles have been depicted in the Fig. S12. An intermediary value of allele frequency for the variant rs1051266 was observed. The allele frequency in the Indian subcontinent comprising of the PJL (Punjabi’s from Lahore, Pakistan), ITU (Indian Telugu from the UK) and STU (Sri Lankan Tamil from the UK) was intermediary, around 0.4 in a range of 0 (low) to 1 (high). Similar allele frequencies were observed in the GIH (Gujaratis Indian from Houston, Texas), BEB (Bengali from Bangladesh), GBR (British in England and Scotland), and CEU (Western European Ancestry) populations. The frequency of the variant rs12190287 for found inclined towards a higher side being around 0.7 for the Indian subcontinent. A similar high frequency was seen for the BEB (Bengali from Bangladesh), MXL (Mexican Ancestry from Los Angeles USA) and PEL (Peruvians from Lima, Peru) populations. The variant rs2298881 had a comparably lower frequency worldwide. Its frequency in India was on a lower side, around 0.2, with similar frequency observed in BEB (Bengali from Bangladesh) and ASW (Americans of African Ancestry in SW USA) populations. However, in the far eastern populations including the JPT (Japanese in Tokyo, Japan) and CHB (Han Chinese in Beijing, China) a higher frequency of these variants was observed. A very high frequency of about 0.8 for the variant 2,229,080 was observed in the Indian population. A similar high frequency of the variant was observed in JPT (Japanese in Tokyo, Japan) and BEB (Bengali from Bangladesh) populations. Similar frequency rates could indicate a higher BC rate in these regions. The wide gap between the genetic frameworks of the different populations makes it essential to analyze the genetic heterogeneity among various populations.

## Conclusion

This is the first study that provided the preliminary data for the J&K population highlighting the role of 15 variants with BC development. Among the studied variants, four variants namely rs1051266, rs12190287, rs2229080 and rs2298881 showed significant association with BC. The logistic regression with age and BMI revealed the best-fit model to be dominant in the case of rs1051266 (*SLC19A1*) and rs12190287 (*TCF21*), while allelic in case of rs2229080 (*DCC*) and additive in the variant rs2298881 (*ERCC1*). These variants displayed maximum effect in the populations when incorporated in these models. The RNA fold analysis revealed the structure variations in wild and altered allele along with the differences in the free energy of the secondary structures. In silico analysis of these variants showed the variants to be evolutionarily conserved thus harboring minimum alterations in them over generations, which enables them to maintain their putative structure and function stability efficiently. Three variants rs1051266, rs12190287 and rs2298881 showed significant eQTL effect. The network analysis gave a deeper insight into the nuanced interactions of the candidate genes with other common proteins, alleging a common pathway of function. The associated loci may affect the development of BC in the women of Jammu and Kashmir and should be further verified in independent data sets. For a better understanding of the gene effect more variants of the genes should be further examined. It might be plausible that there are other variants, which have not been studied and have an eloquent association with BC. Our results provide a clue for further functional validation to reveal underlying genetic mechanisms in BC. These SNPs subsequent to validation can aid in the development of a breast cancer panel specific for the population under study. The specific testing can separate the potential risk targets and early detection could be beneficial in the treatment process. This further can also help in the early detection and personalized medicine for the breast cancer patients.

## Supplementary information

**Additional file 1.**

**Additional file 2.**

## Data Availability

Data and material are available. The datasets generated or analyzed during the current study are not publicly available but are available with the corresponding author and can be provided on reasonable request.

## Abbreviations

BMI: Body Mass Index
CI: Confidence Interval
BC: Breast Cancer
HWE: Hardy Weinberg equilibrium
J&K: Jammu, and Kashmir
NGS: Next generation sequencing
NES: Normalized effect Size
OR: Odds Ratio
PCR: Polymerase chain reaction
SNP: Single nucleotide polymorphism
SAP: Shrimp alkaline phosphatase
PJL: Punjabi’s from Lahore, Pakistan
ITU: Indian Telugu from the UK
STU: Sri Lankan Tamil from the UK
GIH: Gujaratis Indian from Houston, Texas
BEB: Bengali from Bangladesh
GBR: British in England and Scotland
CEU: Western European Ancestry
MXL: Mexican Ancestry from Los Angeles USA
PEL: Peruvians from Lima, Peru
ASW: Americans of African Ancestry in SW USA
JPT: Japanese in Tokyo, Japan
CHB: Han Chinese in Beijing, China
