# Supplementary material for: MassARRAY-based single nucleotide polymorphism analysis in breast cancer of north Indian population
Source: BMC Cancer. 2020 Sep 7;20:861. doi: 10.1186/s12885-020-07361-8 (PMC7487711; doi:10.1186/s12885-020-07361-8)
Supplement: Supplementary file 1 — Additional file 1. [file 12885_2020_7361_MOESM1_ESM.docx]

**Supplementary Files-Tables**

**MassARRAY-based single nucleotide polymorphism analysis in breast cancer of North Indian Population**

**Authors**: Divya Bakshi, MSc^*1^, Ashna Nagpal, MSc^1^, Varun Sharma, PhD^2^, Indu Sharma, PhD^2^, Ruchi Shah, MSc^1^, Bhanu Sharma, MSc^1^, Amrita Bhat, MSc^1^, Sonali Verma, PhD^1^, Gh. Rasool Bhat, MSc^1^, Deepak Abrol, MD^3^, Rahul Sharma, MD^4^, Samantha Vashnavi, PhD^5^, Rakesh Kumar, PhD^*1^

^1^School of Biotechnology, Shri Mata Vaishno Devi University, Katra, India

^2^Birbal Sahni Institute of Paleosciences, Lucknow,UP, India

^3^Department of Radiotherapy, GMC, Kathua, J&K, India

^4^Department of Radiotherapy, GMC, Jammu, J&K, India

^5^Department of Plant Sciences, Central University of Jammu, J&K, India

**Table S1: Putative role of studied genes and SNPs annotation**

| S.No. | GENE NAME | ROLE | VARIANT | SNP ANNOTATION |
| --- | --- | --- | --- | --- |
| 1 | *PALB2* | Tumor suppressor | rs249954 | Intron Variant |
| 2 | *ATM* | DNA damage response | rs664677 | Intron Variant |
| 3 | *FGFR2* | Regulation of cell proliferation | rs2981582 | Intron Variant |
| 4 | *SLC4A7* | Bicarbonate transporter, maintains pH homeostasis | rs4973768 | UTR Variant 3 Prime |
| 5 | *ANKLE1* | Regulation of DNA repair | rs2363956 | Missense, Transcript Variant |
| 6 | *SLC19A1* | Folate transporter | rs1051266 | Missense,Upstream Variant 2KB,UTR Variant 5 Prime |
| 7 | *TCF21* | Tumor suppressor | rs12190287 | UTR Variant 3 Prime |
| 8 | *CYP19A1* | Cell cycle regulation and apoptosis | rs10046 | Intron Variant, UTR Variant 3 Prime |
| 9 | *DCC* | Tumor suppressor gene | rs2229080 | Missense |
| 10 | *ERCC1* | DNA repair | rs2298881 | Intron Variant |
| 11 | *TERT* | Telomere maintenance | rs2736100 | UTR-5 Variant |
| 12 | *TERT* | Telomere maintenance | rs2735940 | Upstream Variant |
| 13 | *TERF1* | Telomere maintenance | rs2975843 | Upstream Variant |
| 14 | *BRIP1* | Tumor suppressor, HR repair | rs4986764 | Downstream Variant 500B, Missense |
| 15 | *REV1* | DNA repair and partner of p53 | rs3792152 | Intron Variant |

**Table S2: Logistic regression analysis of variants of genes in our study, adjusted for age and BMI**

| **GENE NAME** | **VARIANT** | **CHROM**  **OSOME NUMBER** | **MINOR ALLELE** | **F_A** | **P VALUE** | **OR*** | **95% C.I.FOR OR*** |  | **P VALUE** | **OR**** | **95% C.I.FOR OR**** |  | **P VALUE** | **OR**** | **95% C.I.FOR OR**** |  |
| --- | --- | --- | --- | --- | --- | --- | --- | --- | --- | --- | --- | --- | --- | --- | --- | --- |
|  |  |  |  |  |  |  | **L95** | **U95** |  |  | **L95** | **U95** |  |  | **L95** | **U95** |
| SLC19A1 | rs1051266 | 21 | T | 0.4679 | 0.000000466 | 3.461 | 2.136 | 5.609 | 0.147 | 1.548 | 0.858 | 2.791 | 0 | 2.026 | 1.476 | 2.783 |
| TCF21 | rs12190287 | 6 | C | 0.4589 | 0.022 | 1.713 | 1.08 | 2.716 | 0.596 | 1.164 | 0.664 | 2.038 | 0.061 | 1.341 | 0.986 | 1.822 |
| DCC | rs2229080 | 18 | C | 0.3143 | 0.305 | 0.797 | 0.517 | 1.229 | 0.05 | 0.505 | 0.252 | 1.014 | 0.088 | 0.758 | 0.551 | 1.042 |
| ERCC1 | rs2298881 | 19 | A | 0.2128 | 0.035 | 0.669 | 0.46 | 0.973 | 0.111 | 0.354 | 0.099 | 1.268 | 0.035 | 0.669 | 0.46 | 0.973 |
| ANKLE-1 | rs2363956 | 19 | T | 0.3708 | 0.009 | 0.522 | 0.322 | 0.848 | 0.348 | 0.758 | 0.425 | 1.352 | 0.024 | 0.683 | 0.491 | 0.95 |
| ATM | rs664677 | 11 | C | 0.3947 | 0.401 | 1.265 | 0.731 | 2.188 | 0.807 | 0.91 | 0.426 | 1.941 | 0.639 | 1.096 | 0.748 | 1.607 |
| TERT | rs2736100 | 5 | A | 0.3188 | 0.934 | 1.018 | 0.663 | 1.565 | 0.581 | 0.809 | 0.38 | 1.72 | 0.853 | 0.969 | 0.696 | 1.35 |
| PALB2 | rs249954 | 16 | A | 0.3172 | 0.553 | 1.145 | 0.732 | 1.792 | 0.476 | 1.333 | 0.604 | 2.941 | 0.438 | 1.149 | 0.809 | 1.632 |
| FGFR2 | rs2981582 | 10 | A | 0.3448 | 0.269 | 1.27 | 0.831 | 1.94 | 0.74 | 0.882 | 0.42 | 1.852 | 0.477 | 1.126 | 0.812 | 1.559 |
| SLC4A7 | rs4973768 | 3 | T | 0.4062 | 0.227 | 0.722 | 0.427 | 1.224 | 0.531 | 0.815 | 0.431 | 1.544 | 0.249 | 0.811 | 0.568 | 1.158 |
| CYP19A1 | rs10046 | 15 | A | 0.331 | 0.869 | 0.965 | 0.63 | 1.478 | 0.372 | 0.715 | 0.343 | 1.491 | 0.59 | 0.914 | 0.659 | 1.268 |
| TERT | rs2735940 | 5 | G | 0.3071 | 0.349 | 1.229 | 0.799 | 1.891 | 0.097 | 0.343 | 0.097 | 1.214 | 0.888 | 1.027 | 0.714 | 1.476 |
| TERF1 | rs2975843 | 8 | G | 0.4173 | 0.912 | 0.975 | 0.623 | 1.526 | 0.334 | 1.306 | 0.76 | 2.246 | 0.651 | 1.072 | 0.793 | 1.45 |
| BRIP1 | rs4986764 | 17 | G | 0.4255 | 0.409 | 0.822 | 0.517 | 1.308 | 0.408 | 0.787 | 0.447 | 1.387 | 0.306 | 0.848 | 0.619 | 1.163 |
| REV1 | rs3792152 | 2 | A | 0.489 | 0.431 | 1.215 | 0.748 | 1.972 | 0.307 | 1.299 | 0.787 | 2.146 | 0.271 | 1.185 | 0.876 | 1.603 |

*Dominant model corrected with age and BMI

**Recessive model corrected with age and BMI

***Additive model corrected with age and BMI

| Table S3: Putative Role of the associated variants in studied population utilizing the information from the various databases including GTEX and UCSC genome browser  \| **Variant** \| **Location of the variant** \| **Allele Ref/Alt** \| **eQTL gene** \| **eQTL Tissue** \| **eQTL sample size** \| **eQTL NES** \| **eQTL *p*-value** \| **eQTL m-value** \| **Putative role (cis- eQTL) of variant** \| \| --- \| --- \| --- \| --- \| --- \| --- \| --- \| --- \| --- \| --- \| \| rs1051266 \| Missense,Upstream Variant \| C/T \| SLC19A1 \| Breast \| 396 \| -0.433 \| 0.0000024 \| 1 \| Significant and Down regulation \| \| rs12190287 \| UTR Variant 3 Prime \| G/C \| TCF21 \| Breast \| 396 \| 0.21 \| 0.000033 \| 1 \| Significant and Up regulation \| \| rs2229080 \| Exonic \| G/C \| DCC \| Breast \| 396 \| 0.0544 \| 0.3 \| 0 \| Non-Significant \| \| rs2298881 \| Intronic \| C/A \| ERCC1 \| Breast \| 396 \| -2.6 \| 3.8E-09 \| 1 \| Significant and Down regulation \|   **NES – Normalized Effect Size in eQTL; m-value – posterior probability that effect exists in each tissue, ranges between 0 and 1.**  **Table S4: Free energies of the variants** |
| --- | --- | --- | --- | --- | --- | --- | --- | --- | --- | --- | --- | --- | --- | --- | --- | --- | --- | --- | --- | --- | --- | --- | --- | --- | --- | --- | --- | --- | --- | --- | --- | --- | --- | --- | --- | --- | --- | --- | --- | --- | --- | --- | --- | --- | --- | --- | --- | --- | --- | --- |

| **Variant** | **Minimum Free Energy (Kcal/Mol)** | **Free Energy of The Thermodynamic Ensemble (Kcal/Mol)** | **Minimum Free Energy of Centroid Structure (Kcal/Mol)** |
| --- | --- | --- | --- |
| rs12190287 (G) | -301.4 | -316.89 | -234.28 |
| rs12190287 (C) | -301.4 | -317.28 | -222.46 |
| rs1051266 (C) | -461.1 | -480.01 | -372.57 |
| rs1051266 (T) | -459.6 | -478.19 | -340.07 |
| rs2229080 (G) | -228.6 | -248.78 | -141.7 |
| rs2229080 (C) | -231.3 | -251.24 | -164.3 |
| rs2298881 (C) | -372.3 | -386.93 | -277.15 |
| rs2298881 (A) | -373.5 | -388.88 | -308.95 |
