# Supplementary material for: MassARRAY-based single nucleotide polymorphism analysis in breast cancer of north Indian population
Source: BMC Cancer. 2020 Sep 7;20:861. doi: 10.1186/s12885-020-07361-8 (PMC7487711; doi:10.1186/s12885-020-07361-8)
Supplement: Supplementary file 2 — Additional file 2. [file 12885_2020_7361_MOESM2_ESM.docx]

**Supplementary Section**

**MassARRAY-based single nucleotide polymorphism analysis in breast cancer of North Indian Population**

**Authors**: Divya Bakshi, MSc^*1^, Ashna Nagpal, MSc^1^, Varun Sharma, PhD^2^, Indu Sharma, PhD^2^, Ruchi Shah, MSc^1^, Bhanu Sharma, MSc^1^, Amrita Bhat, MSc^1^, Sonali Verma, PhD^1^, Gh. Rasool Bhat, MSc^1^, Deepak Abrol, MD^3^, Rahul Sharma, MD^4^, Samantha Vashnavi, PhD^5^, Rakesh Kumar, PhD^*1^

^1^School of Biotechnology, Shri Mata Vaishno Devi University, Katra

^2^Birbal Sahni Institute of Paleosciences, Luckhnow,UP

^3^Department of Radiotherapy, GMC, Kathua

^4^Department of Radiotherapy, GMC, Jammu

^5^Department of Plant Sciences, Central University of Jammu


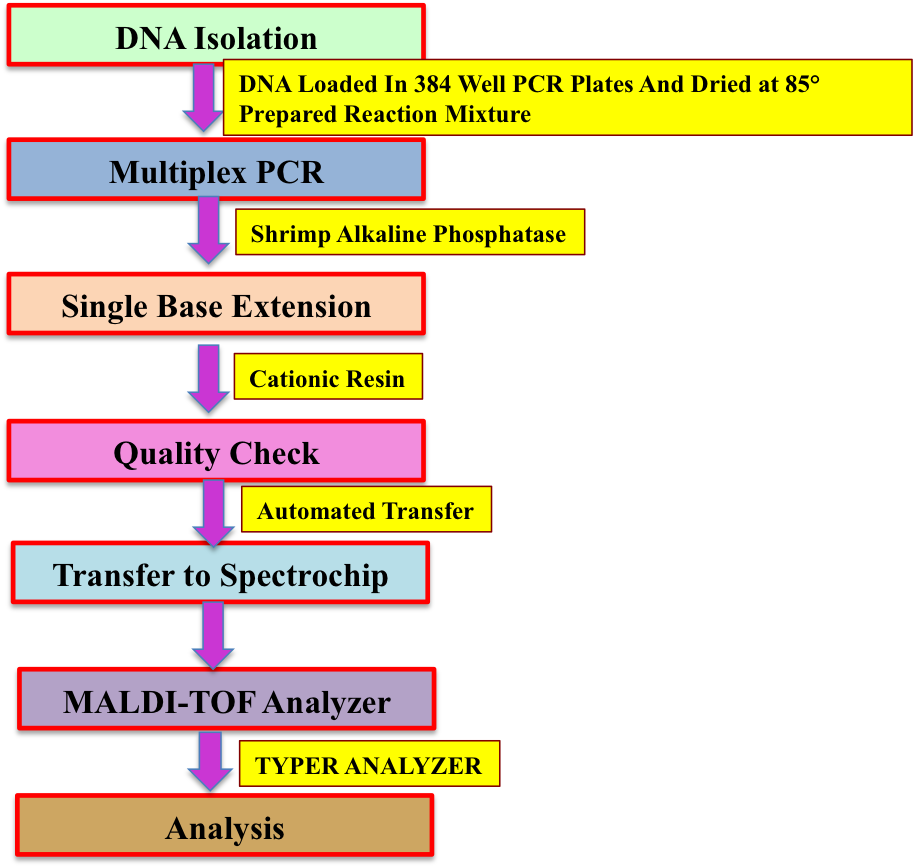


**Figure S1: The workflow of Agena MassArray genotyping**

**Figure S2: Allele frequency in *TCF21* gene Figure S3: Allele frequency in *DCC* gene**

**Figure S4: Allele frequency in *SLC19A1* gene. Figure S5: Allele frequency in *ERCC1* gene**


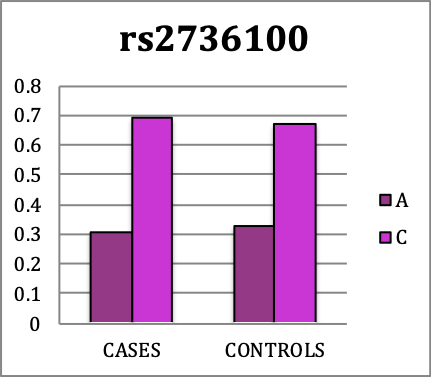

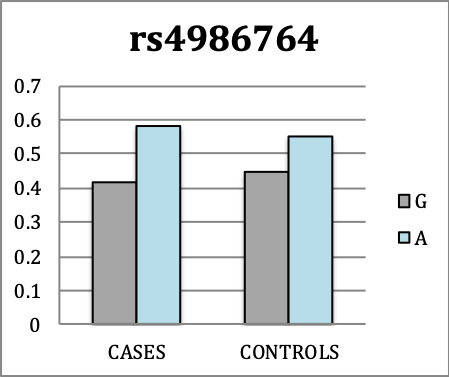


**Figure S6: Allele frequency *in TERT* Figure S7: Allele frequency in BRIP1**


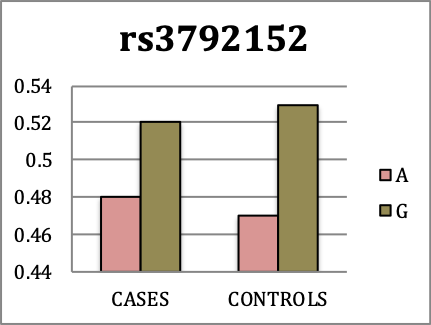


**Figure S8: Allele frequency in *REV1* Figure S9: Allele frequency in *TERF1***

.

**Figure S10: Allele frequency in *FGFR2* Figure S11: Allele frequency in *CYP19A1***


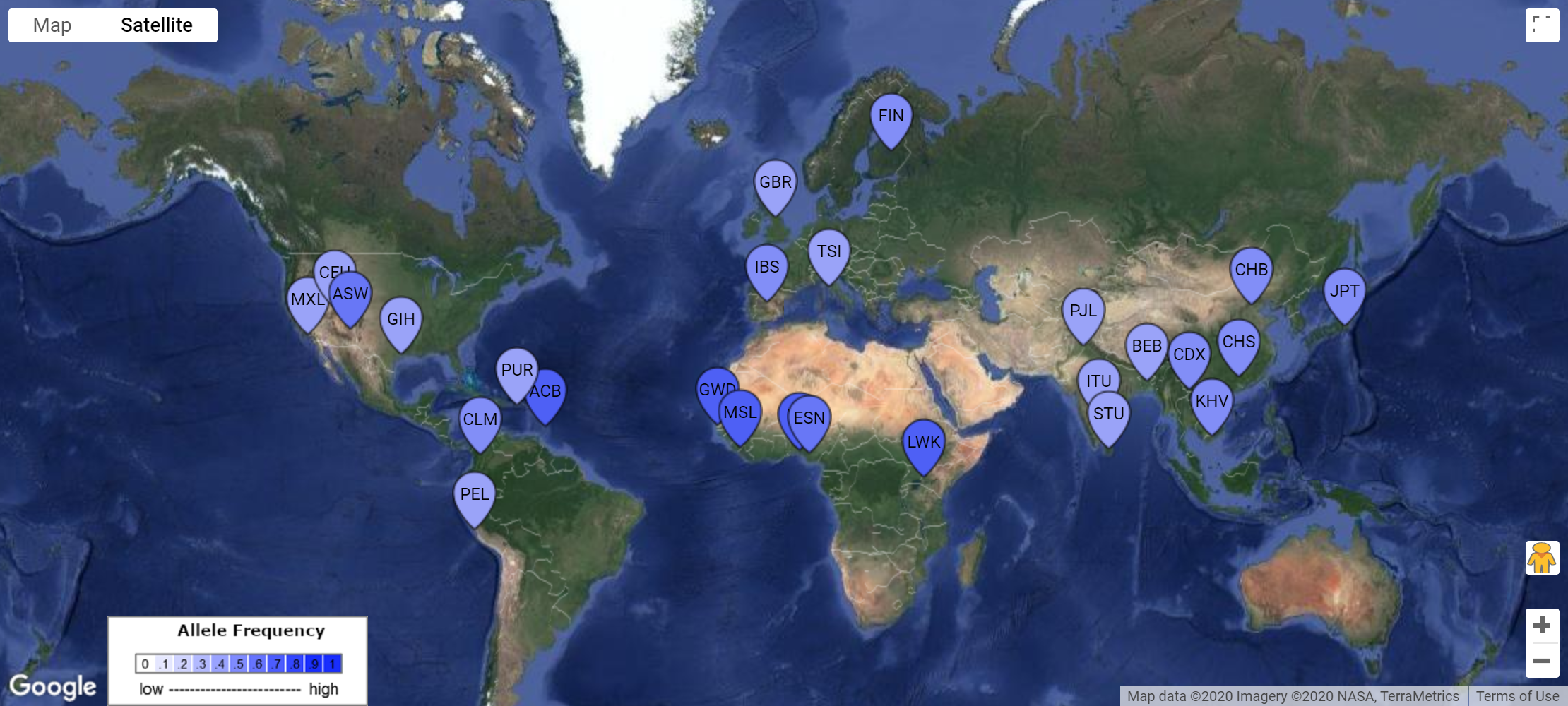

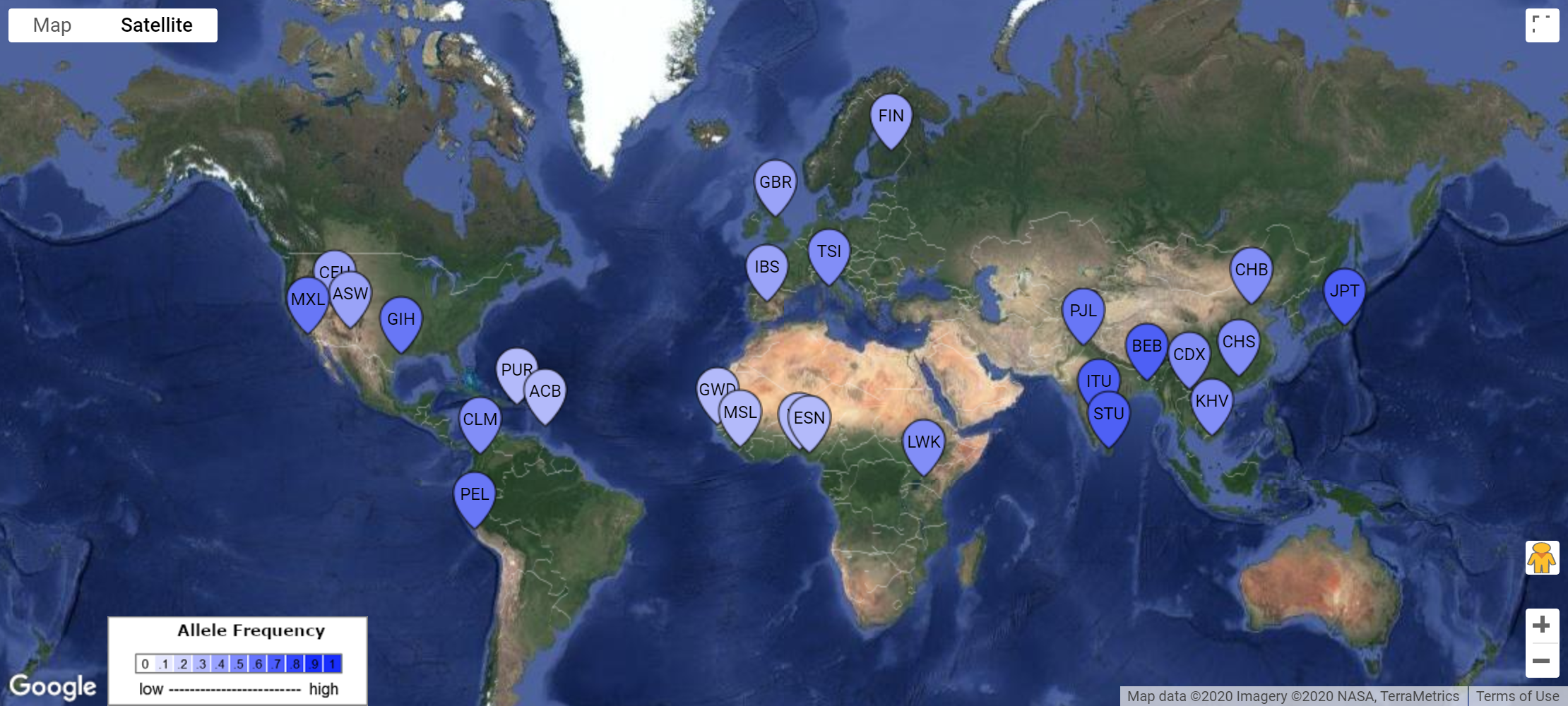

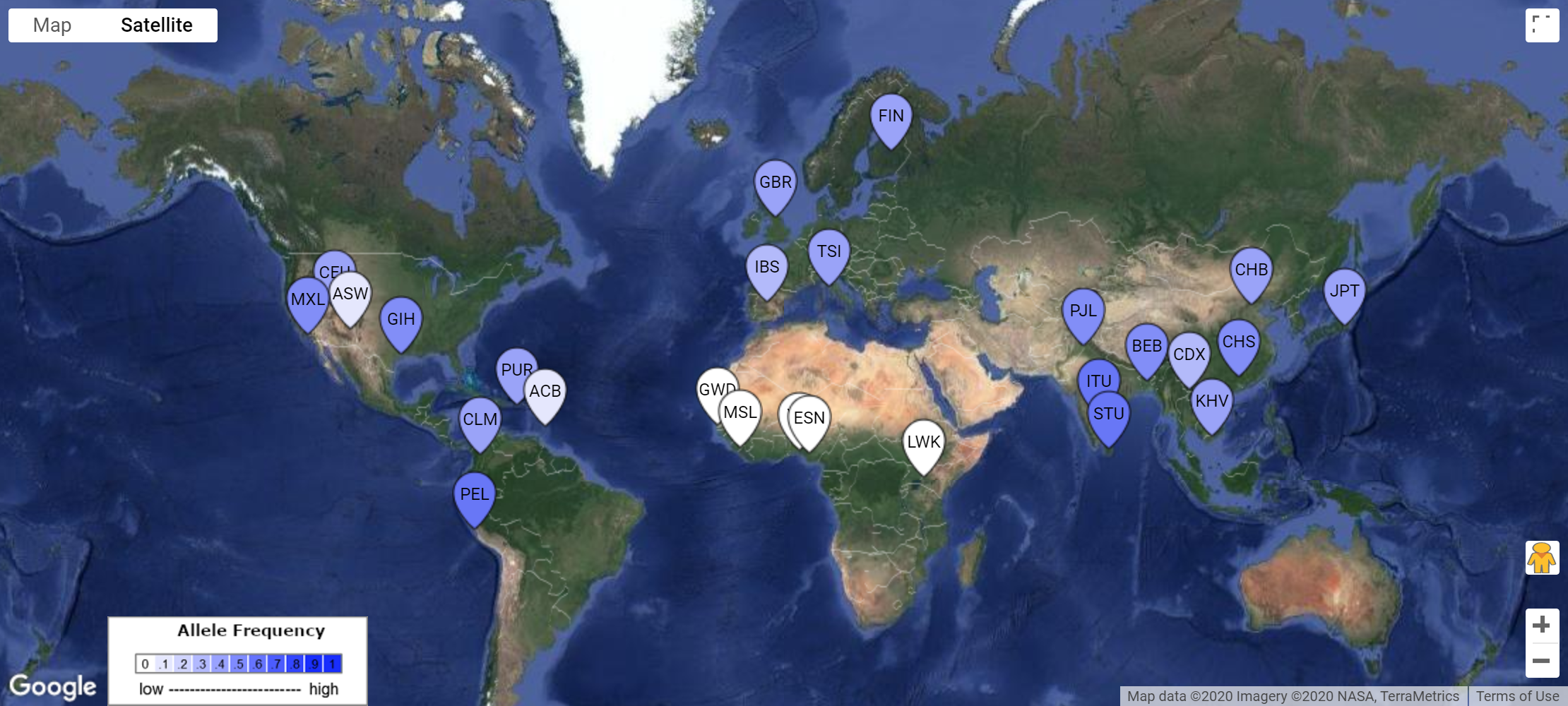

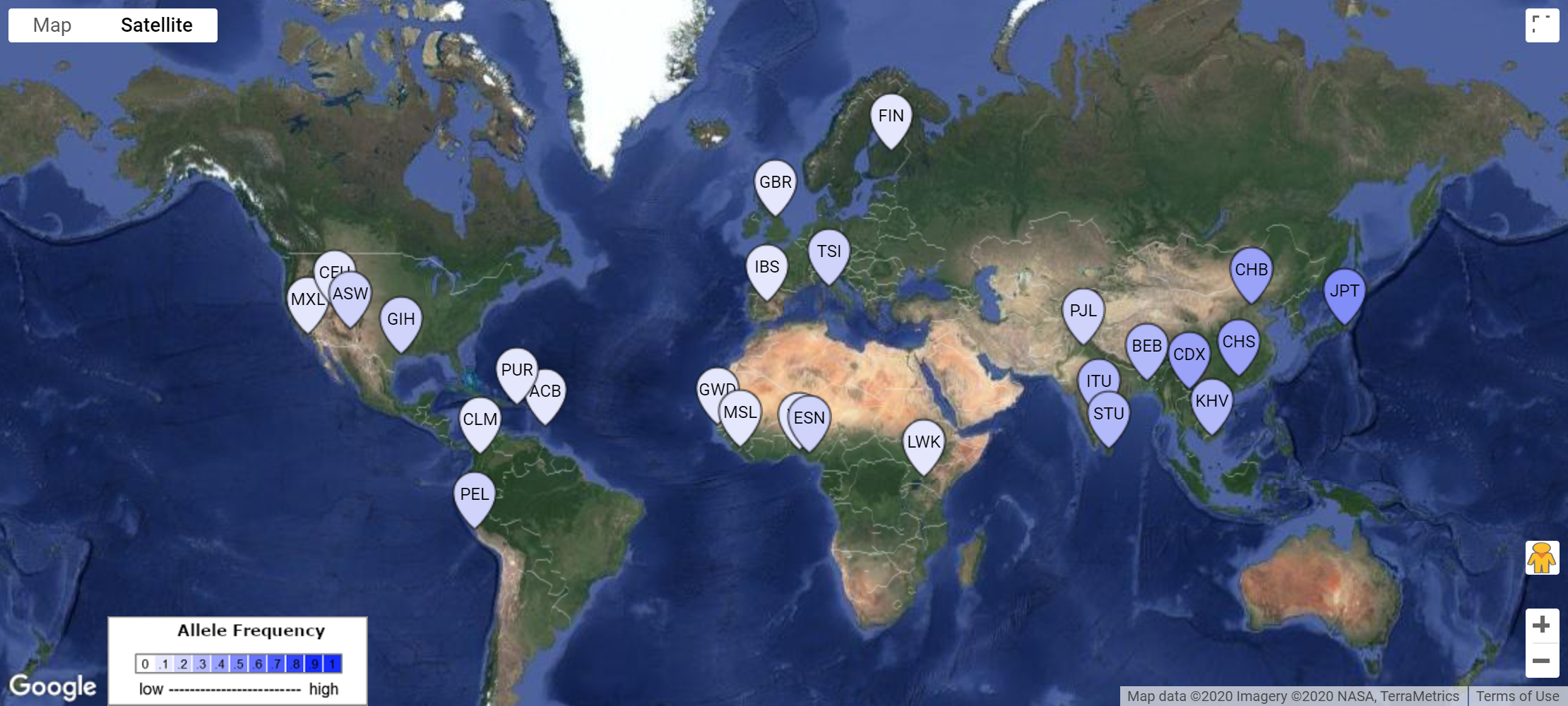


A

D

B

c

**Figure S12: Worldwide allele frequency of the variants rs1051266 (A), rs2229080 (B), rs12190287 (C) and rs2298881 (D) of the *SLC19A1, DCC, TCF21* and *ERCC1* gene compared with 1000 genome data.**

**.**

|         **Figure S13: Screenshot from UCSC Genome Browser representing conservation of the variant nucleotide rs1051266, rs2229080, rs12190287 and rs2298881 of the *SLC19A1, DCC, TCF21* and *ERCC1* gene respectively*,* across 100 vertebrates** |
| --- |
